# Artificial Intelligence Legislation Literacy, Governance Readiness, and Adoption Intentions in Romanian Healthcare: A Cross-Sectional Study

**DOI:** 10.3390/healthcare14131867

**Published:** 2026-06-26

**Authors:** Alina Doina Tănase, Cristian Zaharia, Ștefania Dinu, Camelia-Oana Mureșan, Daliana Emanuela Bojoga, Raluca-Mioara Cosoroabă, Emanuela Lidia Petrescu

**Affiliations:** 1 Department of Professional Legislation in Dental Medicine, Faculty of Dental Medicine, “Victor Babes” University of Medicine and Pharmacy, Eftimie Murgu Square 2, 300041 Timisoara, Romania; tanase.alina@umft.ro (A.D.T.); cosoroaba.raluca@umft.ro (R.-M.C.); 2 Research Centre in Dental Medicine Using Conventional and Alternative Technologies, Faculty of Dental Medicine, “Victor Babes” University of Medicine and Pharmacy, Eftimie Murgu Square 2, 300041 Timisoara, Romania; cristian.zaharia@umft.ro (C.Z.); petrescu.emanuela@umft.ro (E.L.P.); 3 Department of Prostheses Technology and Dental Materials, Faculty of Dental Medicine, “Victor Babes” University of Medicine and Pharmacy, Eftimie Murgu Square 2, 300041 Timisoara, Romania; 4 Pediatric Dentistry Research Center (Pedo-Research), Department of Pediatric Dentistry, Faculty of Dental Medicine, “Victor Babes” University of Medicine and Pharmacy, Eftimie Murgu Square 2, 300041 Timisoara, Romania; 5 Discipline of Forensic Medicine, Bioethics, Deontology, and Medical Law, Department of Neuroscience, “Victor Babes” University of Medicine and Pharmacy, Eftimie Murgu Square 2, 300041 Timisoara, Romania; 6 Ethics and Human Identification Research Center, “Victor Babes” University of Medicine and Pharmacy, Eftimie Murgu Square 2, 300041 Timisoara, Romania; 7 University Clinic of Oral Rehabilitation and Dental Emergencies, Faculty of Dentistry, “Victor Babes” University of Medicine and Pharmacy Timisoara, Eftimie Murgu Square 2, 300041 Timisoara, Romania; mocuta.daliana@umft.ro; 8 Interdisciplinary Research Center for Dental Medical Research, Lasers and Innovative Technologies, Revolutiei 1989 Avenue 9, 300070 Timisoara, Romania

**Keywords:** artificial intelligence, legislation as topic, data protection, diffusion of innovation, health personnel

## Abstract

Background and Objectives: As Romanian health systems deploy artificial intelligence (AI), uptake depends on navigating the EU AI Act, GDPR, the Medical Device Regulation (MDR), and national rules. We measured AI legislation literacy, governance readiness, and adoption intentions among Romanian healthcare professionals, identified implementation phenotypes, and tested whether confidence mediates the literacy–adoption link. Materials and Methods: In a multicenter cross-sectional survey (N = 109), participants completed a 20-item AI Legislation Literacy Index (0–20) plus scales rated form one to five measuring legislative confidence, adoption intention, readiness, trust, and perceived compliance burden. We used PCA and k-means clustering, multivariable logistic regression for high adoption intention (≥4), and covariate-adjusted mediation (5000 bootstrap resamples). Results: Mean age was 38.7 ± 9.8 years, and 60.6% of participants were female. Literacy was moderate (11.2 ± 4.1/20) and familiarity favored GDPR (69.7%) over the EU AI Act (25.7%). Literacy correlated with confidence (=0.52), whereas confidence correlated with adoption intention (=0.41); trust correlated positively (=0.44) and burden correlated negatively (=−0.29) with adoption. High adoption intention was noted in 50.5% of participants and was independently associated with higher literacy (aOR 1.85 per +1 SD; 95% CI 1.20–2.85), higher trust (aOR 1.72; 1.13–2.63), lower burden (aOR 0.64; 0.43–0.95), and prior AI training (aOR 2.10; 1.03–4.29). Three phenotypes emerged (Confident Adopters n = 44; Cautious Compliers n = 36; Skeptical Low Literacy n = 29), with adoption scores of 4.2 ± 0.5 vs. 3.1 ± 0.7 in the highest and lowest groups. Mediation showed a partial indirect effect via confidence (0.13; 95% CI 0.05–0.24). Conclusions: AI legislation literacy, confidence, trust, and perceived burden are key, modifiable determinants of AI adoption intentions; phenotype-guided strategies can target training, governance support, and post-deployment monitoring readiness. The revised framing explicitly situates these determinants within recent AI-specific regulatory and technical developments, including high-risk AI obligations, AI-enabled medical device change control, generative/large multimodal model risks, and lifecycle monitoring.

## 1. Introduction

Artificial intelligence (AI) has moved from experimental decision support to operational use across healthcare workflows, including triage and risk prediction, imaging interpretation, clinical documentation, and administrative automation. As these systems move closer to patient care, regulatory compliance becomes a practical determinant of feasibility, trust, procurement decisions, and institutional liability. In the European Union (EU), the EU AI Act introduces a risk-based legal framework with explicit obligations for high-risk AI systems, many of which are directly relevant to clinical tools because of their potential effects on safety and fundamental rights [1]. Healthcare AI must also satisfy health data requirements such as lawful basis, transparency, purpose limitation, data minimization, and security. Recent legal–technical analyses, therefore, argue that the EU AI Act’s duties should be interpreted together with existing obligations governing health sector data handling and accountability [2]. Legal scholarship in biomedicine similarly shows that compliance is not merely a privacy checkbox, but an ongoing alignment between technical design choices and patient rights protections when AI systems rely on sensitive health data [3]. Even when privacy-enhancing strategies such as federated learning are used, implementation still depends on how organizations operationalize confidentiality, controller/processor roles, cross-border processing, and auditability under data protection law [4]. Many clinical AI tools also intersect with medical device oversight, so differences between regulatory pathways in the USA and Europe can shape local expectations regarding evidence thresholds, post-market change control, and accountability [5]. Critically, these instruments do not operate in isolation but apply concurrently and interact; the same clinical AI tool may simultaneously be a “high-risk” system under the EU AI Act, a regulated medical device under the MDR, and a processor of special category health data under the GDPR. The AI Act explicitly defers to and builds upon existing sectoral law, so its conformity assessment, risk management, and post-market monitoring duties are layered on top of—rather than replacing—MDR clinical evaluation requirements and GDPR lawful basis, transparency, and data minimization obligations. This overlapping architecture creates practical ambiguity for healthcare institutions, which must reconcile three partially distinct compliance logics—product safety (MDR), fundamental rights and algorithmic risk (AI Act), and data protection (GDPR)—within a single procurement and deployment decision, often without consolidated national guidance on how these regimes intersect.

This multi-layer compliance environment creates a practical governance challenge for health systems: institutions must translate legal requirements into procurement criteria, validation plans, human oversight workflows, monitoring, incident response, and documentation. Health system governance models for clinical AI emphasize the need for defined roles (clinical, technical, legal/compliance), standardized evaluation pipelines, and lifecycle monitoring to manage safety and accountability once tools are embedded into care processes [6]. However, implementation science suggests that adoption is rarely driven by “capability” alone. Classical acceptance theories (e.g., the Technology Acceptance Model) show that perceived usefulness and ease of use shape uptake, but these perceptions are frequently moderated in healthcare by risk, professional accountability, and organizational constraints that include regulatory burden [7]. Broader implementation frameworks, such as the Consolidated Framework for Implementation Research (CFIR), highlight that successful deployment depends on intervention characteristics, inner setting readiness, and individual knowledge/beliefs, precisely the domains where legal literacy and compliance confidence may exert measurable effects [8]. Similarly, the NASSS framework (nonadoption, abandonment, scale-up, spread, sustainability) highlights that complexity across the technology, the organization, and the wider system can derail scale-up even when pilot performance appears favorable [9]. In recent evidence syntheses, barriers and facilitators to healthcare AI adoption repeatedly include governance readiness, uncertainty about legal and ethical obligations, perceived workload, and a lack of practical guidance for safe implementation [10,11].

More specifically, five recent developments now define the urgency of implementation-ready compliance literacy: EU high-risk AI obligations, AI-enabled medical device change management, generative and large multimodal models, privacy-preserving data infrastructures, and transparent clinical evaluation standards. Reviews describe the rapid expansion of AI applications across specialties and highlight that clinical translation now hinges on data quality, validation design, workflow integration, and continuous monitoring rather than algorithmic novelty alone [12]. Conceptually, high-performance medicine is increasingly framed as a convergence between human expertise and AI capabilities, but only when systems are deployed in ways that preserve safety, accountability, and trust [13]. Health systems’ experience also shows that delivering clinical impact is often limited by generalizability gaps, dataset shift, unclear performance reporting, integration friction, and misalignment with end-user needs—limitations that intersect with legal duties around transparency, risk management, and post-market surveillance [14]. Methodological critiques likewise caution that strong headline performance may not translate to real-world benefit, and systematic review-level evidence comparing AI performance to clinicians has underscored frequent reporting limitations and risks of bias that can complicate both clinical governance and legal defensibility [15]. At the same time, landmark demonstrations of AI performance in specific diagnostic tasks (e.g., dermatologist-level image classification) illustrate why clinical teams and institutions remain interested in AI adoption, even as they seek clearer guardrails for responsible deployment [16].

Romania provides a particularly relevant setting to study these dynamics. Healthcare delivery spans highly regulated public hospitals, rapidly adopting private providers, and academic centers engaged in research and innovation—structures that may differ substantially in their access to compliance expertise, digital health training, and governance infrastructure. Radiology offers an instructive example of this variability: surveys of specialists show strong expectations that AI will reshape workflows, alongside concerns about responsibility, validation, and preparedness—tensions that likely generalize to other clinical domains and influence implementation behavior [17]. At the same time, recent analyses of authorized AI/ML medical technologies highlight persistent transparency gaps and heterogeneous reporting practices, raising practical questions for hospitals about how to evaluate tools, document due diligence, and sustain post-deployment monitoring [18,19]. In parallel, benefit–risk reporting assessments of FDA-cleared AI/ML devices emphasize that even when products reach market, the information available to support local governance decisions may be inconsistent—reinforcing the importance of internal literacy and structured readiness when institutions make adoption choices [20].

### Specific Recent Developments and Literature Gaps Addressed

The revised literature framing distinguishes concrete developments that are not fully captured by generic AI adoption discussions. First, the EU AI Act shifts the debate from broad ethical principles to operational obligations for high-risk clinical AI, including risk management, technical documentation, data governance, human oversight, logging, accuracy, robustness, cybersecurity, serious incident reporting, and post-market monitoring [1,2,21]. Second, AI-enabled software increasingly functions as medical device software or decision-support infrastructure whose performance may change after deployment, so institutions need local validation, predetermined change control thinking, and monitoring of calibration, drift, and subgroup performance rather than a one-time procurement decision [5,18,19,20,22]. Third, foundation models and large multimodal models are expanding AI use beyond narrow imaging classifiers into documentation, summarization, triage, patient communication, research support, and administrative workflows; these tools introduce distinctive risks, including hallucinated content, leakage of sensitive data, opaque model updating, automation bias, and difficulty in assigning accountability when output is repurposed clinically [23,24,25]. Fourth, privacy-enhancing approaches such as federated learning can reduce direct data sharing but do not remove GDPR obligations related to lawful basis, role allocation, transparency, purpose limitation, data minimization, security, and auditability [3,4]. Fifth, contemporary reporting and evaluation standards increasingly emphasize transparent model development, early-stage clinical evaluation, workflow impact, and human factors reporting, making clinician legal literacy and institutional readiness prerequisites for defensible adoption rather than peripheral concerns [14,15,26,27].

These developments define the gap addressed by the present study. Existing reviews describe barriers, facilitators, trust, explainability, or broad governance models [10,11,28,29,30,31,32,33,34,35], but they rarely measure objective AI legislation literacy alongside confidence, readiness, perceived burden, and adoption intention within the same empirical framework. The current study, therefore, contributes a context-specific empirical link between regulatory knowledge and adoption behavior in Romanian healthcare, where the transition from GDPR-oriented awareness to AI Act- and MDR-oriented operational capability remains underexplored. The analysis also extends prior literature by translating implementation heterogeneity into actionable phenotypes rather than treating healthcare professionals as a single adopter group.

In this cross-sectional study, we quantified AI legislation literacy, legislative confidence, governance readiness, trust, and perceived compliance burden among Romanian healthcare professionals. We also used data-driven clustering to identify implementation phenotypes and tested a mechanistic hypothesis that literacy influences adoption partly through confidence. We hypothesized that (i) higher legal literacy would co-occur with higher confidence and readiness; (ii) perceived compliance burden would inversely relate to adoption intention; and (iii) distinct phenotypes would emerge that may be actionable for policy and training.

## 2. Materials and Methods

### 2.1. Study Design and Setting

We conducted a multicenter, cross-sectional survey to assess AI legislation literacy, governance readiness, and AI adoption intentions among healthcare professionals in Romania. Data were collected from participants affiliated with public hospitals, private clinics, and academic/university settings, reflecting heterogeneous organizational contexts in which clinical AI tools may be procured, validated, and deployed. The survey targeted both end-users (physicians, nurses, and allied health) and governance-facing stakeholders (administrators/managers, legal/compliance, IT/data) because AI implementation requires multidisciplinary accountability across clinical, technical, and regulatory domains. The study was conducted in accordance with the Declaration of Helsinki and followed institutional ethics approval procedures (see Institutional Review Board Statement). Participation was voluntary, anonymous, and uncompensated. Reporting follows standard cross-sectional survey conventions (STROBE-aligned structure).

### 2.2. Participants and Sampling

Eligible participants were adults (≥18 years) employed in healthcare delivery, management, legal/compliance, or health-IT functions within participating institutions. We used convenience sampling with role quotas to ensure representation of clinicians and governance stakeholders. Exclusion criteria were: (i) incomplete consent, and (ii) missing responses preventing computation of the primary outcomes. A total of 116 respondents opened the survey; 109 provided analyzable data (7 excluded: 3 did not consent; 4 had >30% missing items).

### 2.3. Measures

We measured AI legislation literacy using a 20-item AI Legislation Literacy Index (scores range from 0 to 20). Item development followed a three-step process: domain mapping to the EU AI Act, the General Data Protection Regulation (GDPR), the Medical Device Regulation (MDR), and Romanian national implementing measures for data protection; expert review by four academics with expertise in medical law, digital health, and clinical AI; and pilot testing in 12 healthcare professionals to refine wording and response clarity. Each item was scored as correct/incorrect, and higher scores indicated greater objective literacy. Content validity was supported by item-level content validity indices ranging from 0.83 to 1.00 and a scale-level average content validity index of 0.94. Internal consistency of the dichotomous literacy scale was acceptable (Kuder–Richardson 20 = 0.78). Legislative confidence (1–5) assessed self-perceived ability to apply legal requirements in practice (higher = more confident). AI adoption intention (1–5) captured near-term intention to use or support AI in routine workflows. Governance readiness (1–5) captured perceived availability of policies, risk management processes, documentation, and oversight. Trust in AI (1–5) and perceived compliance burden (1–5) were measured using 4-item subscales (mean scores), with acceptable internal consistency (Cronbach’s = 0.81 and 0.76, respectively). Construct validity of the multi-item attitudinal subscales was further supported by the principal component analysis described in Section 2.5, in which items loaded coherently onto interpretable governance dimensions (see Section 3.4), and by the expected pattern of inter-construct correlations (literacy–confidence, confidence–adoption, trust–adoption; Section 3.5), consistent with theoretically predicted convergent and discriminant relationships. The literacy index was scored as a simple unweighted sum of correct responses (range 0–20), with no reverse-coded items; the 1–5 attitudinal subscales were scored as the arithmetic mean of their constituent items after reverse-coding where applicable, so that higher values consistently denote greater confidence, adoption intention, readiness, trust, or perceived burden. 

The survey was administered electronically using a secure web-based platform. After reading an information page, participants provided electronic informed consent before accessing the questionnaire items. The instrument was organized into four logical blocks (demographics/role; literacy items; attitudes; readiness/governance), and pilot administration indicated a completion time of approximately 10–12 min. Mandatory responses were not enforced in order to reduce satisficing; however, participants could review their answers before submission. No identifying information (names, emails, or IP addresses) was retained in the analytic dataset. Responses were exported to a structured dataset for analysis. Quality checks included verification of consent, screening for excessive missingness, range checks, and confirmation that literacy items permitted valid scoring.

### 2.4. Outcomes

The primary outcomes were AI adoption intention (continuous) and high adoption intention (binary: ≥4). Secondary outcomes included literacy, confidence, governance readiness, trust, and compliance burden. For phenotype discovery, we standardized literacy, confidence, readiness, trust, burden, and adoption intention to z-scores.

### 2.5. Statistical Analysis

Continuous variables were reported as mean ± SD or median [IQR] when skewed; categorical variables were reported as n (%), and between-group comparisons were performed using *t*-tests/ANOVA or Mann–Whitney/Kruskal–Wallis, as appropriate. Associations were evaluated using Spearman correlations (ρ). We derived governance dimensions using PCA (KMO/Bartlett checks, Varimax rotation) and computed factor scores. Phenotypes were identified using k-means clustering on standardized features, selecting k based on silhouette and interpretability. We modeled high adoption intention via multivariable logistic regression, including prespecified covariates (age, sex, sector, role, and prior AI training) and key predictors (literacy, confidence, trust, and compliance burden). Mediation analysis tested Literacy → Confidence → Adoption, adjusting for covariates, with 5000 bootstrap resamples. Factor retention in the PCA was guided by a combination of criteria rather than a single rule: the Kaiser criterion (eigenvalues > 1), inspection of the scree plot for an “elbow,” the proportion of cumulative variance explained, and the interpretability and theoretical coherence of the rotated solution. The two retained components each had eigenvalues above unity, together accounted for 62% of the variance, and produced a simple, interpretable structure with no substantial cross-loadings, supporting a two-dimensional governance readiness solution. For the k-means analysis, features were z-standardized before clustering to prevent scale dominance, and the number of clusters was selected by comparing average silhouette widths across candidate solutions (k = 2–5) alongside substantive interpretability; the three-cluster solution offered the most coherent and clinically meaningful separation. Given the modest total sample and the resulting small subgroup sizes, we treat the clustering as exploratory and hypothesis-generating; cluster stability was not formally established through resampling or split-sample replication, and the phenotypes are therefore reported as provisional implementation-oriented profiles rather than as definitive or population-stable adopter typologies.

## 3. Results

### 3.1. Participant Characteristics

A total of 109 participants were included. The cohort was predominantly female (60.6%) with a mean age of 38.7 years. Participants represented diverse roles and sectors (Table 1). The sample (N = 109) was middle-aged overall (38.7 ± 9.8 years) and predominantly female (66/109, 60.6%), with most respondents drawn from public hospitals (57, 52.3%), followed by private clinics (29, 26.6%) and academic settings (23, 21.1%). Clinicians made up the largest share (physicians 33, 30.3%; nurses 28, 25.7%), but governance-facing roles were also represented (administrators/managers 16, 14.7%; legal/compliance 10, 9.2%; IT/data 10, 9.2%). Despite the topic’s practical relevance, only 37.6% reported any prior AI-related training (41/109), while 42.2% reported some AI use in workflow (46/109), suggesting that real-world exposure is present but not yet matched by formal upskilling.

### 3.2. Outcomes by Role

Objective literacy and self-perceived confidence showed a clear role gradient; legal/compliance had the highest literacy (15.8 ± 2.6/20) and the highest confidence (3.9 ± 0.6/5), alongside the strongest readiness (3.5 ± 0.6), yet their adoption intention was comparatively lower (3.4 ± 0.7), consistent with a more cautious, risk-aware stance. IT/data roles combined high literacy (13.9 ± 3.1) with the highest adoption intention (3.9 ± 0.6), while physicians also demonstrated higher literacy (12.3 ± 3.8) and adoption (3.8 ± 0.7). Nurses had the lowest literacy (10.1 ± 3.7) and lower adoption (3.5 ± 0.8), and overall averages remained moderate across constructs (literacy 11.2 ± 4.1; confidence 3.1 ± 0.9; readiness 2.9 ± 0.8; adoption 3.7 ± 0.8), suggesting room for targeted capability building (Table 2).

### 3.3. Familiarity with Regulatory Frameworks

Self-reported familiarity was uneven and strongly skewed toward established data protection regulation; GDPR familiarity was high (76, 69.7%), whereas familiarity with the newer EU AI Act was much lower (28, 25.7%). MDR familiarity was intermediate (51, 46.8%), and familiarity with Romanian GDPR implementing measures (Law 190/2018) was modest (34, 31.2%). This pattern highlights a “GDPR-first” awareness profile, with AI-specific and national operational details lagging behind, which is important because perceived familiarity is explicitly noted as distinct from objective literacy (Table 3).

### 3.4. Governance Readiness Dimensions

The PCA supported a two-dimensional governance structure that separates “organizational process/accountability” from “technical/documentation capability.” Factor 1 was dominated by policy and oversight infrastructure (procurement/use policy loading 0.78; oversight committee 0.74; risk workflow 0.71), while Factor 2 captured lifecycle evidence and documentation requirements (model documentation 0.81; DPIA capability 0.77; validation/monitoring plan 0.70). Adequacy statistics (KMO = 0.79; Bartlett *p* < 0.001) and explained variance (62%) suggest a coherent measurement structure, reinforcing that readiness is not a single construct but a combination of governance “machinery” and technical documentation capacity (Table 4).

### 3.5. Correlation Structure

The strongest observed association was between literacy and legislative confidence (ρ = 0.52, *p* < 0.001), indicating that knowledge and applied self-efficacy reinforce each other. Adoption intention correlated meaningfully with both confidence (ρ = 0.41, *p* < 0.001) and trust (ρ = 0.44, *p* < 0.001), while compliance burden showed a significant inverse relationship with adoption intention (ρ = −0.29, *p* = 0.002). Literacy also related positively to governance readiness (ρ = 0.36, *p* < 0.001), supporting the interpretation that higher literacy aligns with more favorable perceptions of institutional preparedness (Table 5).

### 3.6. Predictors of High Adoption Intention

After adjustment, high adoption intention was independently associated with higher literacy (aOR 1.85 per +1 SD; *p* = 0.005) and higher trust (aOR 1.72; *p* = 0.012), whereas perceived compliance burden was associated with reduced odds (aOR 0.64; *p* = 0.030). Prior AI training was also a significant facilitator (aOR 2.10; *p* = 0.041), suggesting that training is not merely correlated with adoption but remains influential even after accounting for other predictors in the model. In contrast, age, sex, and public sector setting were not significant (e.g., age aOR 0.92 per 10 years; *p* = 0.602), implying that adoption intentions are shaped more by modifiable “capability-and-belief” factors than by basic demographics (Table 6).

### 3.7. Implementation Phenotypes

Clustering revealed three sharply separated profiles (all ANOVA *p* < 0.001) that differed most in adoption intention, confidence, and burden. “Confident Adopters” (n = 44) showed the highest adoption (4.2 ± 0.5), confidence (3.7 ± 0.6), readiness (3.3 ± 0.6), and trust (3.8 ± 0.5), with comparatively lower burden (3.5 ± 0.6). “Cautious Compliers” (n = 36) were intermediate in adoption (3.4 ± 0.6) but had the highest compliance burden (4.1 ± 0.5), suggesting willingness tempered by perceived workload/risk. “Skeptical Low Literacy” (n = 29) had the lowest literacy (8.4 ± 3.8), confidence (2.3 ± 0.7), readiness (2.3 ± 0.6), trust (2.9 ± 0.6), and adoption (3.1 ± 0.7), indicating a group likely to benefit most from foundational training and structured support. Because clustering was exploratory and some professional strata were small, these profiles should be interpreted as implementation-oriented heuristics rather than fixed adopter classes (Table 7).

### 3.8. Mediation Analysis

Mediation results supported a partial indirect pathway in which literacy increases confidence (path a: β = 0.45, *p* < 0.001), and confidence in turn increases adoption intention (path b: β = 0.29, *p* = 0.002). Literacy also retained a direct association with adoption intention even after accounting for confidence (c′: β = 0.18, *p* = 0.010), consistent with partial (not full) mediation. The bootstrapped indirect effect was 0.13 with a 95% CI of [0.05, 0.24], reinforcing that improving literacy may raise adoption intentions partly by strengthening users’ perceived ability to operationalize legal requirements, while also exerting additional direct influence. Because all variables were measured concurrently in a single cross-sectional, convenience-sampled survey of modest size (N = 109), this mediation should be interpreted as statistical decomposition of association consistent with a plausible mechanism rather than as evidence of a causal or temporal pathway; the same data are compatible with reverse or reciprocal influences (for example, adoption intention shaping confidence), and the indirect effect, though statistically distinguishable from zero, is small in absolute terms. We therefore frame the literacy → confidence → adoption pathway as hypothesis-generating, to be confirmed in longitudinal or experimental designs (Table 8).

**Figure 1:**
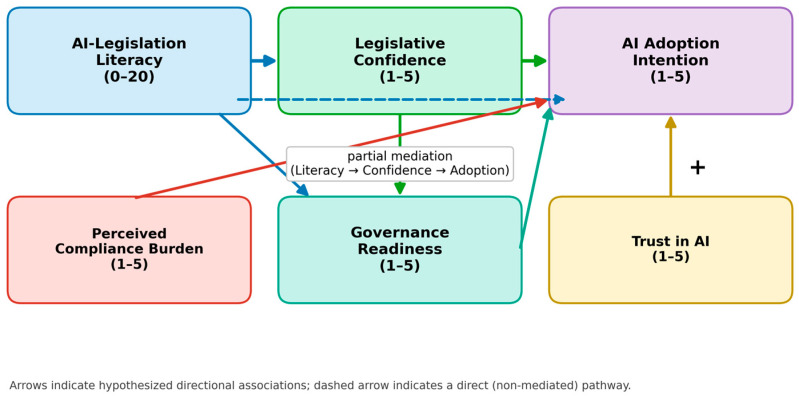
Framework linking AI legislation literacy, legislative confidence, governance readiness, trust, perceived compliance burden, and AI adoption intention.

The proposed framework is supported by several key associations observed: literacy correlated with legislative confidence (ρ = 0.52, *p* < 0.001), and confidence correlated with adoption intention (ρ = 0.41, *p* < 0.001). In adjusted modeling, higher literacy predicted high adoption intention (aOR 1.85 per +1 SD, *p* = 0.005), while perceived compliance burden reduced the odds (aOR 0.64 per +1 SD, *p* = 0.030), and trust increased them (aOR 1.72 per +1 SD, *p* = 0.012). Mediation results align with this structure, showing a partial indirect pathway via confidence (indirect effect = 0.13; 95% CI 0.05–0.24), as seen in Figure 1.

**Figure 2:**
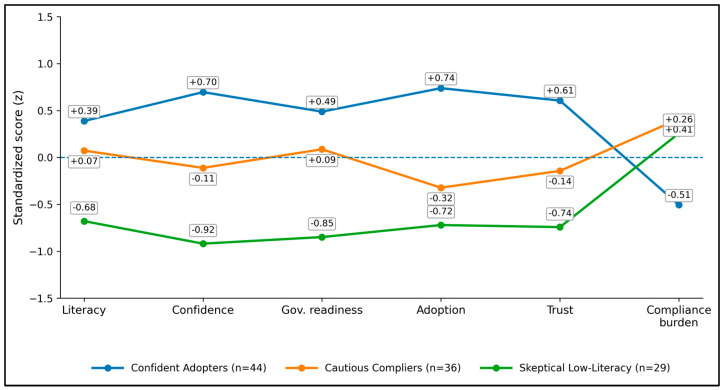
Cluster-derived implementation phenotypes: standardized signatures.

The three phenotypes differ most in adoption intention, confidence, and compliance burden. “Confident Adopters” (n = 44) had the highest adoption intention (4.2 ± 0.5) and confidence (3.7 ± 0.6) with lower burden (3.5 ± 0.6), whereas “Skeptical Low Literacy” (n = 29) showed lower adoption (3.1 ± 0.7) and confidence (2.3 ± 0.7) with higher burden (4.0 ± 0.7). “Cautious Compliers” (n = 36) were intermediate for adoption (3.4 ± 0.6) but had the highest burden (4.1 ± 0.5). Literacy also separates groups (12.8 ± 3.6 vs. 8.4 ± 3.8), consistent with the standardized signatures (Figure 2).

## 4. Discussion

### 4.1. Analysis of Findings

Across Romanian settings, we observed moderate objective AI legislation literacy (11.2 ± 4.1/20) alongside a marked familiarity gradient (GDPR 69.7% vs. EU AI Act 25.7%). This pattern is consistent with evidence that data-protection awareness is more mature than AI-specific governance preparedness, including Romanian public-sector experience where GDPR implementation has been described as uneven, resource-constrained, and strongly dependent on local compliance capacity [36]. Similarly, international clinician surveys across multiple specialties show broad enthusiasm for AI’s potential but persistent uncertainty around regulation, accountability, and practical “rules of the road,” which likely depresses AI Act–specific familiarity when legal requirements are newly introduced or perceived as complex [37]. Taken together, our results suggest that knowledge gaps are not simply “digital skills” gaps; rather, they reflect differential exposure to compliance workflows (e.g., DPIAs, procurement documentation, post-deployment monitoring) and the extent to which institutions operationalize these requirements into routine practice. This low EU AI Act familiarity (25.7%) should be interpreted critically in light of the Act’s phased implementation calendar rather than as simple professional disengagement. The Regulation entered into force in 2024, but its obligations apply progressively—prohibited practice provisions and AI literacy duties first, followed by governance and general purpose model rules, with the bulk of the high-risk obligations most relevant to clinical AI not becoming fully applicable until 2026–2027. At the time of data collection, much of the high-risk framework therefore remained prospective rather than operational, which plausibly explains why awareness lagged well behind the long-established, fully enforceable GDPR. Read this way, the GDPR–AI Act familiarity gap is less a static knowledge deficit than a predictable feature of a regulatory transition still in progress—and a time-limited window in which targeted, anticipatory literacy-building could pre-empt the compliance pressure that will accompany full applicability of the high-risk regime.

A key contribution of this study is the mechanistic structure linking literacy → confidence → adoption intention. Literacy correlated strongly with legislative confidence (ρ = 0.52), and confidence correlated with adoption intention (ρ = 0.41), with mediation supporting a partial indirect pathway (indirect effect 0.13; 95% CI 0.05–0.24). Prior work indicates that clinicians and trainees are often receptive to clinical AI yet report limited practical knowledge, and that greater understanding is associated with more favorable attitudes and implementation readiness [38]. Institutional readiness research likewise emphasizes that training and organizational change support are central levers for shifting readiness from abstract optimism to operational use, which aligns with our finding that prior AI training doubled the odds of high adoption intention (aOR 2.10) [39]. Mixed-methods evidence further shows that physicians’ acceptance hinges on perceived benefits, but also on transparency, role clarity, and concrete integration frameworks—elements that plausibly map onto “confidence” as the proximal determinant of action in high-accountability clinical environments [40].

The observed literacy differences appear to be patterned by professional role, institutional environment, and prior exposure to digital health technologies rather than distributed at random. By role, objective literacy followed a clear gradient, with legal/compliance (15.8 ± 2.6) and IT/data staff (13.9 ± 3.1) having the highest scores and nurses the lowest (10.1 ± 3.7), mirroring the extent to which each role engages directly with regulatory and data governance tasks in daily practice. Institutional context is also plausibly relevant: our sample spanned public hospitals, private clinics, and academic centers, which differ substantially in access to compliance expertise, dedicated data-protection officers, procurement governance, and structured digital health training—differences that can shape the routine workflows through which legal knowledge is acquired and reinforced. Prior exposure appeared influential as well, because only 37.6% of respondents reported any prior AI-related training despite 42.2% reporting some AI use in workflow, and prior training remained an independent predictor of high adoption intention after adjustment (aOR 2.10). Together, these patterns suggest that literacy is not a fixed individual trait but is co-determined by occupational function, organizational infrastructure, and cumulative hands-on experience with digital tools. Because subgroup sizes for several roles were small and the design was cross-sectional, these role- and setting-level differences should be regarded as descriptive and hypothesis-generating; nonetheless, they reinforce the case for role-tailored and institution-specific literacy interventions rather than for uniform training delivered identically across all professional groups and care settings.

Beyond knowledge, trust emerged as a major independent correlate of high adoption intention (aOR 1.72 per +1 SD), reinforcing that governance must address not only compliance “checks,” but also beliefs about safety, reliability, and institutional stewardship. A recent systematic review on explainable AI (XAI) highlights a crucial nuance: explanations can increase or decrease clinicians’ trust depending on the format, cognitive load, and whether they align with clinical reasoning, implying that “more transparency” is not automatically “more trust” [28]. In parallel, an integrative review of AI acceptance in hospitals identifies recurring determinants at the individual and organizational levels—perceived usefulness, workflow fit, competence/training, leadership support, and concerns about autonomy—supporting our interpretation that trust functions as a composite construct shaped by both technical features and the surrounding implementation environment [29]. In our sample, the “Confident Adopters” phenotype combined higher trust (3.8 ± 0.5) with higher readiness (3.3 ± 0.6), suggesting that trust is reinforced when institutions visibly invest in governance capacity rather than placing responsibility solely on end-users. That trust operated as an independent predictor even after adjusting for literacy, confidence, and perceived burden warrants closer interpretation in terms of transparency, explainability, and ethical governance. Trust in clinical AI is best understood as warranted rather than blind; it is calibrated by whether a system’s behavior, limitations, and provenance can be scrutinized, not merely by surface-level confidence in its outputs. Transparency—encompassing disclosure of training data characteristics, intended use, performance across relevant subgroups, and known failure modes—provides the evidentiary basis on which clinicians can decide whether reliance is justified. Explainability contributes when it is matched to the clinical decision and the user’s reasoning; as the XAI evidence indicates, poorly designed or cognitively burdensome explanations can erode rather than build trust, so explainability should be treated as a means of supporting appropriate reliance and enabling error detection rather than as an end in itself. Ethical governance closes the loop by assigning clear accountability for oversight, validation, incident response, and redress, signaling to clinicians that responsibility for AI-assisted decisions is shared institutionally rather than displaced onto the individual at the point of care. Framed this way, the independent contribution of trust suggests that adoption strategies should pair technical transparency and fit-for-purpose explanations with visible ethical governance structures, since trust that is not anchored in genuine accountability and contestability risks becoming either misplaced over-reliance or defensive avoidance.

The inverse association between perceived compliance burden and high adoption intention (aOR 0.64 per +1 SD) helps explain the “Cautious Compliers” profile (highest burden: 4.1 ± 0.5) and the paradox that legal/compliance roles, despite having the highest literacy (15.8 ± 2.6), showed comparatively lower adoption intention (3.4 ± 0.7). Health system survey data indicate that organizations pursuing AI adoption consistently report friction in governance, resourcing, and implementation processes—domains that translate directly into perceived burden at the clinician/manager level [30]. Legal scholarship on AI in practice also warns that clinicians may become “liability sinks,” absorbing accountability for AI-assisted decisions without proportional control over model design, validation evidence, or monitoring—an issue that can rationally inflate perceived compliance burden and suppress adoption enthusiasm even among AI supporters [31]. Complementing this, systematic review work on medical liability in AI-supported diagnostics underscores persistent uncertainty across responsibility allocation, standards of care, and evidentiary expectations, reinforcing our interpretation that burden is not merely paperwork—it is perceived risk plus workload, especially under unclear accountability regimes [32].

Finally, our phenotype approach (Confident Adopters; Cautious Compliers; Skeptical Low Literacy) offers an actionable bridge between implementation science and compliance strategy. Contemporary organizational case studies show that building AI governance capacity typically requires formal structures (multidisciplinary oversight, procurement pathways, validation/monitoring playbooks, documentation standards) and iterative learning loops to manage drift and post-deployment risk—features that align closely with our PCA-derived readiness dimensions (process/accountability vs. technical/documentation) [33]. Enterprise-scaling guidance similarly argues that governance is the central risk-mitigation mechanism enabling safe scale-up across multiple AI solutions, rather than a single model-by-model effort [34]. Qualitative implementation research using NASSS also emphasizes that adoption success depends on socio-technical complexity (technology, workflow, organization, and wider system) and that “one-size” implementation strategies often fail—supporting our conclusion that stratified interventions are likely needed: (i) targeted literacy/confidence training for Skeptical Low Literacy groups, (ii) burden reduction tooling and standardized templates (DPIA checklists, model documentation bundles) for Cautious Compliers, and (iii) scale-up pathways with monitoring and feedback for Confident Adopters [35]. Viewed through the lens of healthcare workforce adaptation, these phenotypes can be read as differing positions along a continuum from active assimilation to principled or anxious resistance, rather than as fixed personality types. Confident Adopters resemble early adapting professionals who integrate new tools into their working identity and may act as local champions, but who also risk automation bias and over-reliance if scale-up outpaces oversight. Cautious Compliers are particularly informative for workforce adaptation: their willingness coexists with the highest perceived compliance burden, suggesting a form of conditional engagement in which adoption is gated by workload, accountability concerns, and the adequacy of institutional support—a profile prone to disengagement or “malicious compliance” if governance demands are perceived as unsupported or punitive. The Skeptical Low Literacy group most clearly embodies resistance rooted in low confidence and limited readiness rather than ideological opposition; their resistance is plausibly protective, signaling unmet needs for foundational knowledge, psychological safety, and low-friction support before more autonomous use is reasonable. Importantly, resistance in this framing is not simply a barrier to be overcome but a source of governance signals: skepticism concentrated among less prepared staff can flag inadequate training infrastructure, while cautious compliance can flag misaligned workload and accountability structures. Phenotype-matched change management—championing and structured scale-up for adopters, burden reduction and shared accountability for compliers, and confidence building, mentoring, and incremental exposure for skeptics—may therefore support durable workforce adaptation more effectively than uniform mandates, which tend to harden resistance in precisely the groups most in need of support.

Our phenotype framework also has implications beyond initial adoption because organizational readiness must be sustained after deployment. Healthcare AI systems can deteriorate when patient mix, workflow patterns, documentation practices, or clinical standards change, meaning that data drift, concept drift, and calibration drift become operational rather than purely technical problems. In this context, the two readiness dimensions identified by PCA map closely onto the infrastructure needed for lifecycle management: process/accountability capacity supports escalation pathways, incident review, and threshold setting, whereas technical/documentation capacity supports dataset versioning, performance surveillance, and change traceability. From this perspective, Confident Adopters may be suitable for locally supervised pilots with predefined monitoring triggers, Cautious Compliers may benefit from centrally managed monitoring templates and external audit support, and Skeptical Low Literacy settings may require simpler dashboards plus vendor- or network-supported monitoring before more autonomous use is attempted [36,37,38,39,40].

The following observations on feedback loops and reinforcement learning from human feedback extend beyond our survey data and are offered as broader conceptual implications and an explanatory analogy rather than as empirical conclusions drawn from the present study. The mediating role of confidence further suggests that post-deployment learning loops should not be conceived only as safety mechanisms, but also as educational interfaces. Structured clinician feedback on incorrect outputs, borderline cases, unexpected workflow effects, and evolving clinical patterns could simultaneously improve models and strengthen users’ perceived competence. This logic resembles reinforcement learning from human feedback (RLHF), in which human preferences and corrections are used to refine model behavior over time [41,42]. In healthcare, phenotype-matched feedback design may be especially important: Confident Adopters may supply richer proactive feedback suitable for iterative optimization, Cautious Compliers may respond better to structured prompts embedded in governance workflows, and Skeptical Low Literacy users may need low-friction interfaces, worked examples, and immediate support so that feedback activity builds confidence rather than adding burden [41,42].

These lifecycle considerations are also relevant to compliance. The EU AI Act requires post-market monitoring for high-risk AI systems and expects providers to maintain mechanisms for collecting, documenting, and acting on performance and safety information throughout the system life cycle [1,21]. Our readiness dimensions may therefore be interpreted as pragmatic baseline indicators of an organization’s ability to operationalize legally required monitoring loops. A phenotype-matched deployment strategy—aligning monitoring intensity, feedback workflows, and governance support with local readiness—could help healthcare systems meet regulatory expectations while allocating implementation resources proportionately.

Although our survey did not directly measure exposure to generative systems, the rapid diffusion of generative AI and large multimodal models (LMMs) into healthcare warrants explicit consideration as a broader conceptual implication of our findings, because these tools materially widen the governance and literacy demands that the present study set out to characterize. Unlike narrow, task-specific classifiers, generative and multimodal systems are general-purpose, operate over free text, images, and structured data simultaneously, and are increasingly embedded in documentation, summarization, triage support, patient communication, and administrative workflows. This breadth introduces distinctive risks: fabrication of plausible but incorrect content (“hallucination”), inadvertent disclosure or memorization of sensitive health data, sensitivity to prompt phrasing, opaque and frequent model updates that complicate version control, automation bias when fluent outputs are over-trusted, and diffuse accountability when a single model is repurposed across many clinical and non-clinical tasks [23,24,25]. The World Health Organization’s guidance on LMMs similarly emphasizes risks spanning accuracy, bias, data protection, and the difficulty of assigning responsibility across developers, deployers, and clinical users [23]. These characteristics interact directly with our central constructs: they raise the literacy threshold required for safe use (since users must understand not only legal duties but also model failure modes), they place additional weight on trust calibration and transparency, and they amplify perceived compliance burden by blurring the boundary between a discrete “medical device” and a continuously evolving general-purpose tool. Conceptually, therefore, the GDPR-first, AI Act-lagging literacy profile we observed is likely to be most consequential precisely for these generative tools, where regulatory classification, oversight, and post-deployment control are least settled. The breadth of clinical applications now spanning AI—from breast cancer detection and risk prediction to deep learning prognostic models for cervical cancer metastasis and recurrence—further underscores why literacy and governance must keep pace with a rapidly expanding application landscape rather than with a single use case [43,44].

Building on this, post-deployment monitoring readiness and lifecycle governance deserve fuller treatment as the operational core of continuous compliance, again framed as a conceptual extension of our readiness findings rather than a direct empirical result. Under the EU AI Act, obligations for high-risk AI do not end at market entry: providers and deployers must sustain post-market monitoring, logging and record keeping, human oversight, and serious-incident reporting, and must keep technical documentation and risk management current across the system life cycle [1,21]. For adaptive or frequently updated systems, including generative and multimodal models, this implies an ongoing duty to detect and respond to data drift, concept drift, and calibration drift, to revalidate after material changes, and to monitor subgroup performance for emergent inequities. Our two readiness dimensions map onto the infrastructure that continuous compliance requires: the process/accountability dimension underpins escalation pathways, incident review, oversight committees, and change control governance, whereas the technical/documentation dimension underpins dataset versioning, performance surveillance, and traceability of model updates. Interpreted as baseline capability indicators, low readiness on either dimension signals a concrete compliance exposure rather than merely an organizational shortfall, because the same capacities that enable safe lifecycle management are those that the Act expects institutions to demonstrate on an ongoing basis. This reframes governance readiness from a one-time procurement checklist to a standing, auditable capability and suggests that institutions with predominantly Skeptical Low Literacy or Cautious Complier profiles may face disproportionate difficulty meeting continuous compliance obligations unless monitoring is centrally supported, templated, and embedded in routine workflows.

To make the revised literature synthesis explicit, Table 9 maps each contemporary AI development to a concrete implementation challenge and to the empirical construct evaluated in this study.

### 4.2. Study Limitations

This cross-sectional, convenience-sampled survey cannot establish causality or predict real-world behavior beyond self-reported intention; associations (including mediation) should therefore be interpreted as supportive of a plausible mechanism rather than definitive proof. Several constructs (trust, burden, and readiness) were self-reported and may have been influenced by social desirability, institutional culture, or role-based expectations, and the literacy instrument—although content-reviewed and internally consistent—cannot capture the full nuance of legal interpretation in practice. The total sample size was modest, and some subgroups, particularly legal/compliance and IT/data roles, were small, which limits precision for role-level estimates and constrains the stability of cluster-derived phenotypes. These phenotype analyses should therefore be viewed as hypothesis-generating rather than population-stable categories. The Romania-specific setting is a strength for contextual relevance, but it also limits transferability to jurisdictions with different regulatory maturity, procurement pathways, and medico–legal environments. Finally, the study assessed adoption intentions rather than observed implementation outcomes. Future longitudinal, multicenter, and cross-national studies should test whether improving literacy, confidence, and governance infrastructure leads to measurable changes in real-world AI uptake, post-deployment monitoring performance, and compliance capability.

## 5. Conclusions

In Romanian healthcare, AI adoption intentions appear to be shaped by objective AI legislation literacy, confidence in operationalizing legal requirements, trust in AI systems, and perceived compliance burden. These findings extend conventional technology acceptance models by showing that regulatory literacy and governance readiness are not peripheral contextual variables; they are central implementation determinants in clinically accountable environments. In practice, the identified phenotypes support targeted action: foundational literacy building and hands-on support for Skeptical Low Literacy users, burden reduction tools and standardized compliance workflows for Cautious Compliers, and structured scale-up with feedback and monitoring loops for Confident Adopters. In the EU healthcare context, such phenotype-matched strategies may also help institutions align adoption with post-market monitoring, documentation, and accountability expectations under the EU AI Act. Because this was a cross-sectional Romanian survey with a modest sample, the findings should be generalized cautiously. Future research should validate these phenotypes in larger and more diverse cohorts and test whether interventions that improve literacy, confidence, and governance infrastructure produce measurable gains in real-world uptake and lifecycle oversight. The main novelty of the revised manuscript is therefore not the generic observation that AI adoption depends on trust, but the demonstration that AI legislation literacy, legal confidence mediation, and governance phenotypes can be measured together in a country-specific EU implementation setting.

## Figures and Tables

**Table 1:** Participant characteristics (N = 109).

Characteristic	Value
Age, years	38.7 ± 9.8
Female sex	66 (60.6%)
Sector: Public hospital	57 (52.3%)
Sector: Private clinic	29 (26.6%)
Sector: Academic/University	23 (21.1%)
Profession: Physicians	33 (30.3%)
Profession: Nurses	28 (25.7%)
Profession: Allied health	12 (11.0%)
Profession: Administrators/Managers	16 (14.7%)
Profession: Legal/Compliance	10 (9.2%)
Profession: IT/Data	10 (9.2%)
Prior AI-related training (any)	41 (37.6%)
Reported AI use in workflow (any)	46 (42.2%)

**Table 2:** Main outcomes by professional role.

Role	n	Literacy (0–20)	Confidence (1–5)	Governance Readiness (1–5)	Adoption Intention (1–5)
Physicians	33	12.3 ± 3.8	3.1 ± 0.8	2.9 ± 0.8	3.8 ± 0.7
Nurses	28	10.1 ± 3.7	2.9 ± 0.8	2.7 ± 0.7	3.5 ± 0.8
Allied health	12	10.7 ± 3.9	3.0 ± 0.9	2.8 ± 0.8	3.6 ± 0.7
Administrators/Managers	16	11.5 ± 4.3	3.2 ± 0.8	3.0 ± 0.7	3.7 ± 0.8
Legal/Compliance	10	15.8 ± 2.6	3.9 ± 0.6	3.5 ± 0.6	3.4 ± 0.7
IT/Data	10	13.9 ± 3.1	3.4 ± 0.7	3.2 ± 0.7	3.9 ± 0.6
Overall	109	11.2 ± 4.1	3.1 ± 0.9	2.9 ± 0.8	3.7 ± 0.8

**Table 3:** Familiarity with key legal frameworks (self-reported).

Framework	Familiar (n, %)
EU Artificial Intelligence Act	28 (25.7%)
General Data Protection Regulation (GDPR)	76 (69.7%)
Medical Device Regulation (MDR)	51 (46.8%)
Romanian Law no. 190/2018 (GDPR implementing measures)	34 (31.2%)

Note: Familiarity reflects self-report and is not equivalent to objective literacy.

**Table 4:** Governance readiness PCA (Varimax-rotated loadings; illustrative).

Item	Factor 1: Process/Accountability	Factor 2: Technical/Documentation
Clear internal policy for AI procurement/use	0.78	0.22
Defined accountability/oversight committee	0.74	0.19
Documented risk management workflow	0.71	0.31
Clinical validation and monitoring plan	0.36	0.70
Data protection impact assessment capability	0.29	0.77
Model documentation (data, performance, drift)	0.21	0.81

Note: KMO = 0.79; Bartlett *p* < 0.001; two-factor solution explains 62% variance.

**Table 5:** Spearman correlations among key measures.

Pair	ρ	*p*-Value
Literacy vs. Confidence	0.52	<0.001
Confidence vs. Adoption intention	0.41	<0.001
Trust vs. Adoption intention	0.44	<0.001
Compliance burden vs. Adoption intention	−0.29	0.002
Literacy vs. Governance readiness	0.36	<0.001

**Table 6:** Multivariable logistic regression predicting high adoption intention (≥4).

Predictor	aOR (95% CI)	*p*-Value
Literacy (per +1 SD)	1.85 (1.20–2.85)	0.005
Trust (per +1 SD)	1.72 (1.13–2.63)	0.012
Compliance burden (per +1 SD)	0.64 (0.43–0.95)	0.030
Prior AI training (yes vs. no)	2.10 (1.03–4.29)	0.041
Age (per 10 years)	0.92 (0.68–1.25)	0.602
Female sex (vs. male)	1.08 (0.54–2.16)	0.831
Public sector (vs private/academic)	0.88 (0.44–1.78)	0.721

Note: Model adjusted for sector and professional role.

**Table 7:** Data-driven implementation phenotypes (cluster profiles).

Variable	Profile 1: Confident Adopters (n = 44)	Profile 2: Cautious Compliers (n = 36)	Profile 3: Skeptical Low Literacy (n = 29)	*p*
Literacy (0–20)	12.8 ± 3.6	11.5 ± 3.9	8.4 ± 3.8	<0.001
Confidence (1–5)	3.7 ± 0.6	3.0 ± 0.7	2.3 ± 0.7	<0.001
Governance readiness (1–5)	3.3 ± 0.6	3.0 ± 0.7	2.3 ± 0.6	<0.001
Adoption intention (1–5)	4.2 ± 0.5	3.4 ± 0.6	3.1 ± 0.7	<0.001
Trust (1–5)	3.8 ± 0.5	3.3 ± 0.6	2.9 ± 0.6	<0.001
Compliance burden (1–5)	3.5 ± 0.6	4.1 ± 0.5	4.0 ± 0.7	<0.001

Note: Profiles derived using k-means clustering on standardized measures; *p*-values from ANOVA.

**Table 8:** Covariate-adjusted mediation: Literacy → Confidence → Adoption intention.

Path	Estimate (β)	SE	*p*	95% CI/Bootstrap CI
a: Literacy → Confidence	0.45	0.07	<0.001	—
b: Confidence → Adoption	0.29	0.09	0.002	—
c′: Literacy → Adoption (direct)	0.18	0.07	0.010	—
c: Literacy → Adoption (total)	0.31	0.08	<0.001	—
Indirect effect (a × b), bootstrap (B = 5000)	0.13	—	—	[0.05, 0.24]

Note: Outcome = Adoption intention (1–5). Covariates: age, sex, sector, role, prior AI training; illustrative values.

**Table 9:** Reviewer-requested synthesis of specific AI developments, implementation challenges, and relevance to the present study.

Specific Development	Implementation Challenge	Relevance to This Study
EU AI Act high-risk framework	Translating provider/deployer obligations into human oversight, logging, risk management, incident reporting, and post-market monitoring.	Explains why EU AI Act familiarity was low (25.7%) despite higher GDPR familiarity and why compliance burden influenced adoption intention.
AI-enabled medical device software and adaptive AI	Local validation, version control, drift surveillance, subgroup performance monitoring, and change control documentation are required beyond procurement.	Reflected in the PCA dimension separating process/accountability from technical/documentation readiness.
Foundation models and large multimodal models	Hallucination, automation bias, prompt/data leakage, opaque updates, and unclear accountability broaden governance needs beyond imaging or narrow prediction tools.	Supports inclusion of trust, confidence, training, and perceived burden as distinct adoption determinants.
Privacy-enhancing/federated data infrastructures	Reduced data movement does not eliminate GDPR duties related to lawful basis, transparency, purpose limitation, data minimization, security, and auditability.	Justifies measuring objective AI legislation literacy rather than relying only on self-reported familiarity.
Transparent reporting and early clinical evaluation standards	Hospitals need evidence on model development, validation, workflow effects, human factors, and lifecycle monitoring before routine deployment.	Supports phenotype-guided training and readiness interventions rather than a one-size-fits-all adoption strategy.
Liability and accountability uncertainty	Clinicians may be asked to act on AI outputs without proportional control over model design, evidence, updates, or monitoring.	Helps explain the Cautious Compliers profile and the lower adoption intention among high-literacy legal/compliance respondents.

## Data Availability

The data presented in this study are available on request from the corresponding author.
